# Assessing the potency of vitamin D augmentation on saccharometric indices in type 2 diabetes: a meta-analysis

**DOI:** 10.3389/fnut.2025.1677686

**Published:** 2026-01-07

**Authors:** Su Gong, Li Chen

**Affiliations:** Department of Clinical Nutrition, Zhuzhou City Central Hospital, Zhuzhou, Hunan, China

**Keywords:** vitamin D enhancement, saccharometric indices, type 2 diabetes, glucose metabolism, meta-analysis

## Abstract

**Background:**

This meta-analytical investigation sought to elucidate the therapeutic efficacy and tolerability of vitamin D supplementation in modulating glycemic indices among individuals diagnosed with type 2 diabetes (T2D).

**Methods:**

Systematic searches were performed across PubMed, Embase, Web of Science, Cochrane Library, and Chinese databases (CNKI and Wanfang) to identify relevant randomized controlled trials (RCTs). Studies were deemed eligible if they enrolled adult T2D populations, administered vitamin D interventions, and reported outcomes including glycated hemoglobin (HbA1c), homeostasis model assessment of insulin resistance (HOMA-IR), or serum 25-hydroxyvitamin D [25(OH)D] concentrations.

**Results:**

Ten RCTs, encompassing a total of 3,460 participants, met inclusion criteria. Intervention durations varied between 3 and 12 months, and dosing regimens ranged from 500 IU and 60,000 IU. Pooled analysis revealed a significant increase in serum 25(OH)D levels following supplementation (SMD: 4.01; 95% CI: 2.43 to 5.59; *p* < 0.001; I^2^ = 99.3%). In contrast, no statistically significant improvements were observed in HbA1c (SMD: 0.08; 95% CI: −0.25 to 0.37; *p* < 0.001; I^2^ = 86.5%) or HOMA-IR (SMD: 5.95; 95% CI: −2.87 to 14.77; *p* < 0.001; I^2^ = 100.0%). Adverse event profiles were similar between the vitamin D and control groups (RR: 1.19; 95% CI: 0.79 to 1.80; *p* = 0.183; I^2^ = 43.5%).

**Conclusion:**

Vitamin D supplementation yield a consistent rise in serum 25(OH)D concentrations but exerts no discernible effect on glycemic regulation or insulin sensitivity in T2D populations. The favorable safety profile supports its tolerability. Considerable heterogeneity in metabolic outcomes underscores the need for future investigations stratified by baseline vitamin D status, enhancement regimen, and study duration.

## 1 Introduction

The hallmark of Type 2 diabetes (T2D) is characterized by impaired insulin action and the progressive decline of pancreatic β-cell function, ultimately leading to chronic hyperglycemia (1). The global prevalence of T2D has risen dramatically, with 463 million adults affected in 2019, and projections indicating an increase to 700 million by 2045 (2). Individuals with T2D are at heightened risk for a range of complications, particularly cardiovascular disease, which remains the leading cause of morbidity and mortality in this population (3). Recent research has increasingly focused on the potential role of vitamin D in modulating glucose homeostasis and cardiovascular health. Traditionally recognized for its role in calcium metabolism and bone health, vitamin D is now understood to exert wide-ranging effects across multiple physiological systems (4). Numerous observational studies have reported an inverse association between circulating levels of 25-hydroxyvitamin D (25(OH)D) concentrations and the risk of developing T2D and cardiovascular disease (5, 6). Several mechanisms have been proposed to explain these associations. Vitamin D may enhance insulin sensitivity by promoting the expression of insulin receptors and glucose transport proteins in target tissues (7), as well as improve pancreatic *β*-cell performance and insulin secretion via both direct and indirect mechanisms (8). Furthermore, its anti-inflammatory and immune-modulating properties may help mitigate the chronic low-grade inflammation commonly seen in T2D and atherosclerosis (9). These promising findings have prompted numerous randomized controlled trials (RCTs) to evaluate the therapeutic potential of vitamin D supplementation in T2D. However, the results have been inconsistent, with some studies reporting significant improvements in glycemic control and cardiovascular risk markers, while others have found no notable benefit (10, 11). These discrepancies may be attributable to differences in study design, cohort size, vitamin D posology, baseline vitamin D status, and intervention duration. The clinical relevance of these outcomes lies in their direct association with diabetes complications and long-term cardiovascular risk. Moreover, whether vitamin D supplementation exerts differential effects in specific subpopulations—such as those with baseline vitamin D deficiency—remains an open question, underscoring the need for a stratified analysis. This meta-analysis aims to synthesize current evidence to provide clinicians and researchers with a clearer understanding of the efficacy of vitamin D supplementation in T2D management, thereby informing both clinical practice and future research.

## 2 Methods

### 2.1 Literary reconnaissance protocol

A comprehensive literature search was conducted across multiple databases, including PubMed, Embase, Web of Science, Cochrane Library, and Sino-centric scholarly databases like CNKI and Wanfang. The search strategy combined standardized Medical Subject Headings (MeSH) with relevant keywords related to vitamin D and type 2 diabetes. Equivalent search strategies were tailored to the indexing systems of each database. To ensure thoroughness, studies in both English and Chinese were included. No restrictions were placed on publication date. The search covered all available records from the inception of each database through June 15, 2024. A predefined protocol for this meta-analysis was registered in the PROSPERO international prospective register of systematic reviews (Registration ID: CRD420251183459).

### 2.2 Study eligibility criteria

Studies were considered eligible for inclusion if they met the following criteria: (1) randomized controlled experimental design; (2) adult participants (≥18 years) with confirmed type 2 diabetes; (3) intervention protocol employing vitamin D supplementation (regardless of formulation or dosage); (4) comparison against placebo or absence of intervention; and (5) reportage of at least one crucial outcome measure: primary endpoints (HbA1c, HOMA-IR, or serum 25(OH)D levels) or secondary endpoints (adverse events or other clinically relevant outcomes pertaining to diabetes management or vitamin D supplementation).

Studies were excluded if they: (1) involved subjects with type 1 diabetes or gestational diabetes; (2) employed vitamin D analogues or combined vitamin D with confounding interventions; (3) utilized non-randomized or uncontrolled methodologies; or (4) had an intervention duration briefer than 3 months, as shortened periods may prove inadequate for observing meaningful alterations in glycemic control, particularly HbA1c, which reflects average glucose levels over a 2–3 month timeframe.

### 2.3 Article screening and data extraction methodology

Two reviewers independently conducted an initial screening of titles and abstracts to identify studies for potential inclusion. Full-text articles of studies deemed relevant were retrieved and evaluated against the eligibility criteria. Any discrepancies between reviewers were through consensus or adjudicated by a third reviewer.

Data extraction was carried out independently by both reviewers using a harmonized, pre-defined data extraction template. Extracted variables included: lead author, publication year, geographical origin, study architecture, sample size, participant characteristics (age, gender distribution, baseline 25(OH)D status), intervention specifics (vitamin D formulation and dosage, administration duration), details of the comparator group, outcome measures, and any reported adverse events.

### 2.4 Methodological quality appraisal

To appraise the methodological rigor of the included trials, the Jadad Scale was employed (12). This validated instrument evaluates three critical dimensions of randomized controlled trial design: randomization procedure, blinding methodology, and account of participant attrition. Each facet contributes up to 2 points, yielding a cumulative score ranging from 0 to 5, with higher values reflecting greater methodological robustness. In addition to the Jadad Scale, the risk of bias for each included study was assessed using the Cochrane Risk of Bias tool (RoB 2.0) to evaluate potential biases arising from the randomization process, deviations from intended interventions, missing outcome data, outcome measurement, and selection of reported results.

### 2.5 Outcome parameters

Primary outcome measures comprised modifications in HbA1c, HOMA-IR, and serum 25(OH)D levels. Secondary endpoints included the frequency and typology of adverse event.

### 2.6 Statistical analysis framework

All quantitative syntheses were performed using Stata version 16.0 (StataCorp, College Station, TX, USA). For continuous variables, standardized mean discrepancies (SMD) with concomitant 95% credence intervals (CI) were estimated, whereas dichotomous outcomes were analyzed using risk ratios (RR) with 95% CI were computed. A random-effects model was adopted to accommodate anticipated inter-study variability.

Heterogeneity was quantified using the I^2^ statistic, with values of 25, 50, and 75% interpreted as indicative of minimal, moderate, and substantial heterogeneity, respectively (13). To further explore the substantial heterogeneity observed in the primary outcomes, pre-specified subgroup analyses were planned based on intervention duration (≤6 months vs. >6 months), vitamin D dosage (low: <2000 IU/day vs. high: ≥2000 IU/day), and baseline vitamin D status (deficient vs. non-deficient), provided sufficient data were available. Sensitivity analyses were also conducted by excluding studies with Jadad scores ≤2 to assess the robustness of the pooled results.

The potential for publication bias was explored through funnel plots asymmetry and formally tested via Egger’s regression analysis in instances where at least four studies contributed to a given outcome (14). Statistical significance was defined as a *p* value < 0.05.

Given the potential influence of baseline vitamin D status on the metabolic response to supplementation, we attempted to perform a subgroup analysis stratified by baseline serum 25(OH)D levels (e.g., <20 ng/mL vs. ≥20 ng/mL). However, this was limited by the inconsistent reporting of baseline levels and varying definitions of deficiency across the included studies.

## 3 Results

### 3.1 Study selection

**Figure 1:**
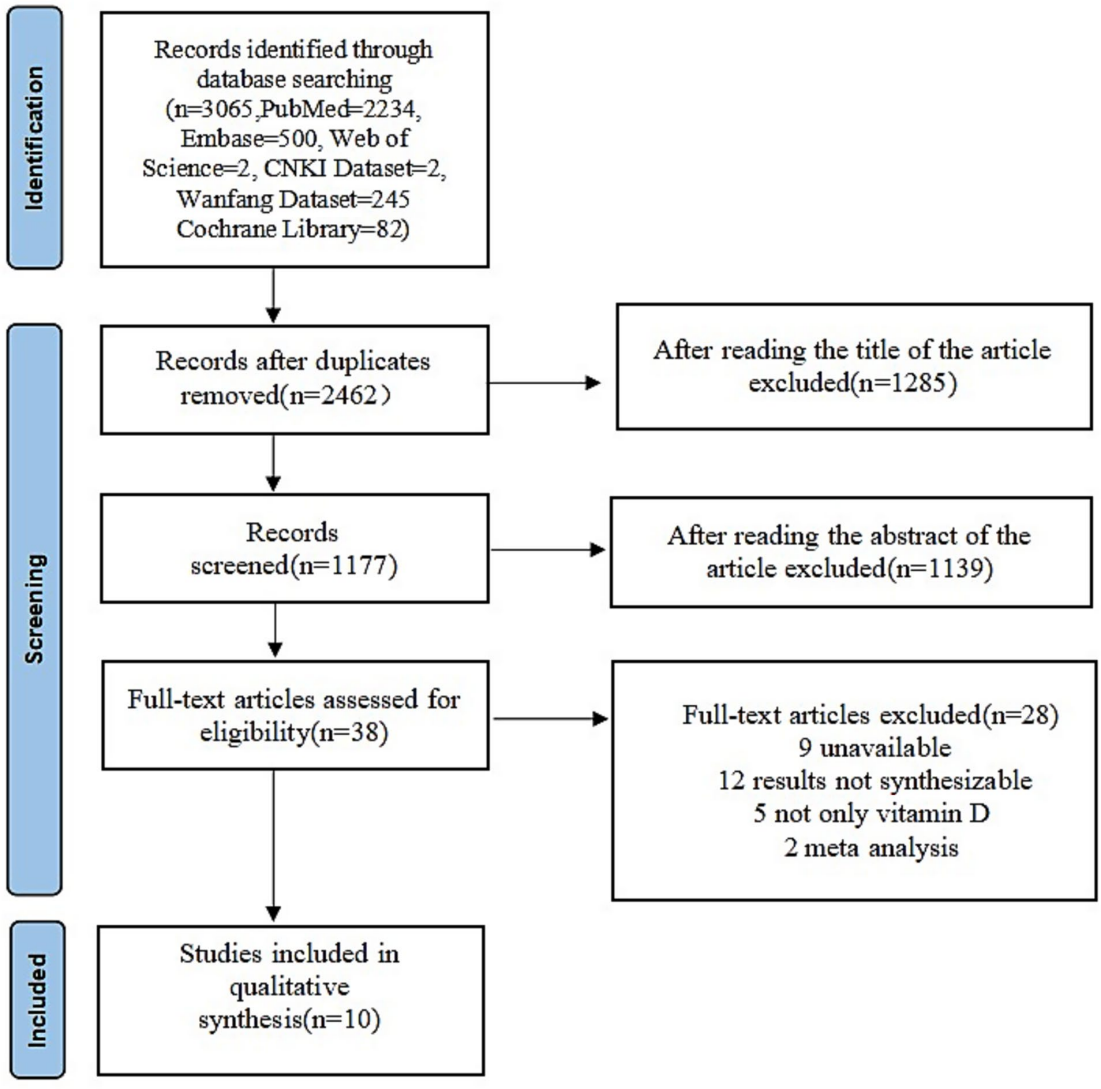
Flow diagram of study selection process.

The systematic search strategy retrieved 3,065 records of potential relevance. After the exclusion of 603 duplicates, 2,462 unique articles proceeded to title screening, which led to the elimination of 1,285 records. Abstracts of the remaining 1,177 studies were reviewed, yielding 38 articles for full-text examination. Ultimately, 10 randomized controlled trials (RCTs) fulfilled all eligibility criteria and were included in the final meta-analysis (15–24). Figure 1 provides a flowchart detailing the selection process.

### 3.2 Study characteristics

Table 1 provides a detailed overview of the constituent randomized controlled trials. In total, 10 studies encompassing 3,460 individuals diagnosed with type 2 diabetes patients were included in the meta-analysis. Sample sizes demonstrated considerable variability, with enrollment figures ranging from 30 to 2,423 participants. The reported mean age across studies extended from 18 to over 30 years. Intervention periods ranged from 3 to 12 months, reflecting modest heterogeneity in follow-up periods.

**Table 1:** Characteristics of the selected studies included in the systematic review and meta-analysis.

Author(year)	Type of study	Treatment measures	Sample size (invention/control)	Age (years)	Outcome indicators	Treatment time	Jadad Scale
Cojic M 2021 (15)	Randomized control trial	50,000 IU of vitamin D3 weekly/14000 IU weekly (4 drops daily)	130(65/65)	≥30	Homeostasis model of assessments; glycemic parameters (FBG, HbA1c); malondialdehyde (MDA); advanced oxidation protein products (AOPP)	3，6 months	3
Byrn M 2019 (16)	Randomized control trial	5,000 IUs weekly	30(15/15)	55.71	Social Adjustment Scale Self-Report (SAS-SR); Self Care Inventory (SCI-R); Problem Areas in Diabetes (PAID)	3，12 months	4
Pittas A 2019 (17)	Randomized control trial	4,000 IU per day	2,423(1,211/1212)	≥30	New-onset diabetes; adverse events	3，6 months	4
Ryu O 2014 (18)	Randomized control trial	2,000 IU per day	81(40/41)	30–69	25-hydroxyvitamin D [25(OH)D] levels; homeostasis model of assessment [HOMA]-IR	6 months	3
Shab-Bidar S 2012 (19)	Randomized control trial	500 IU/250 mL twice a day	100(50/50)	≥30	highly sensitive C-reactive protein (hsCRP), serum amyloid A (SAA), interleukin(IL)-2, IL-6, IL-10 and tumor necrosis factor (TNF)-α	3 months	3
Angellotti E 2018 (20)	Randomized control trial	4,000 IU/day	127(66/61)	≥30	plasma 25-hydroxyvitamin D [25(OH)D] level; lipid profile, C-reactive protein	12 months	3
Johny E 2022 (21)	Randomized control trial	60,000 IU cholecalciferol/week	59(30/29)	35–65	the serum levels of IL-18, TNF-α, IFN-γ, CXCL-10, CXCL-12, CCL-2, CCL-5, CCL-11, and PF-4 levels; vitamin-D-binding protein (VDBP)	6 months	4
Yiu YF 2013 (22)	Randomized control trial	5,000 IU/day	100(50/50)	≥30	Low-mediated dilatation (FMD); endothelial progenitor cells (EPCs); high-sensitivity C-reactive protein (hsCRP)	3 months	3
Forouhi N 2016 (23)	Randomized control trial	20,000 IU per ml/week	340(170/170)	30–75	25-hydroxyvitamin D [25(OH)D]2 level; HbA1c	6 months	2
Witham M 2010 (24)	Randomized control trial	20,000 IU/week	70(35/35)	18	Endothelial function, office blood pressure, B-type natriuretic peptide, insulin resistance and glycosylated haemoglobin	2,3 months	3

The vitamin D supplementation strategies demonstrated considerable heterogeneity across trials. Three studies employed daily dosing regimens: 2,000 IU (18), 4,000 IU (20), and 5,000 IU (22). while five adopted weekly regimens, including: 5,000 IU (16), alternating between 14,000 IU (as 4 daily drops) and 50,000 IU (15), 20,000 IU (24), 60,000 IU (21), and 20,000 IU per mL (23). One study opted for twice-daily dose of 500 IU/250 mL (19), while another administered 4,000 IU per day (17). Cholecalciferol (vitamin D3) was the predominant form employed across all interventions.

Methodological quality, assessed via the Jadad Scale, ranged from 2 to 4 out of 5. Notably, eight studies attained scores of 3 or higher, denoting robust trial design, whereas two studies received a score of 2, suggestive of moderate methodological soundness.

Table 2 summarizes the baseline characteristics of the included studies, with a particular focus on the mean baseline serum 25(OH)D levels of the participants. The reported baseline levels varied across studies, with several populations exhibiting vitamin D insufficiency (e.g., <20 ng/mL or 50 nmol/L) at the outset. However, inconsistent reporting and varying thresholds for defining deficiency limited a unified analysis of this moderator.

**Table 2:** Baseline vitamin D status and key moderators for subgroup analysis.

Author (Year)	Baseline 25(OH)D (Intervention)	Baseline 25(OH)D (Control)	Vitamin D deficiency threshold	Dosage category	Duration category	Baseline deficiency reported
Cojic M 2021 (15)	44.6% < 50 nmol/L	43.1% < 50 nmol/L	<50 nmol/L	High	Medium-Long	Yes
Byrn M 2019 (16)	67.3 nmol/L (Mean)	65.8 nmol/L (Mean)	Not Specified	Medium	Medium-Long	No
Pittas A 2019 (17)	28.1 ng/mL (Mean)	28.3 ng/mL (Mean)	<12 ng/mL	High	Medium-Long	Yes
Ryu O 2014 (18)	15.3 ng/mL (Mean)	15.1 ng/mL (Mean)	<20 ng/mL	Medium	Medium	Yes
Shab-Bidar S 2012 (19)	34.1 nmol/L (Mean)	35.3 nmol/L (Mean)	<50 nmol/L	Low	Short	Yes
Angellotti E 2018 (20)	22.5 ng/mL (Mean)	23.1 ng/mL (Mean)	<20 ng/mL	High	Long	Yes
Johny E 2022 (21)	18.7 ng/mL (Mean)	19.2 ng/mL (Mean)	<20 ng/mL	High	Medium	Yes
Yiu YF 2013 (22)	24.3 nmol/L (Mean)	25.1 nmol/L (Mean)	<50 nmol/L	High	Short	Yes
Forouhi N 2016 (23)	27.9 nmol/L (Mean)	28.5 nmol/L (Mean)	<30 nmol/L	High	Medium	Yes
Witham M 2010 (24)	18.9 nmol/L (Mean)	19.5 nmol/L (Mean)	<50 nmol/L	High	Short	Yes

### 3.3 Primary outcomes

#### 3.3.1 Glycated hemoglobin (HbA1c)

**Figure 2:**
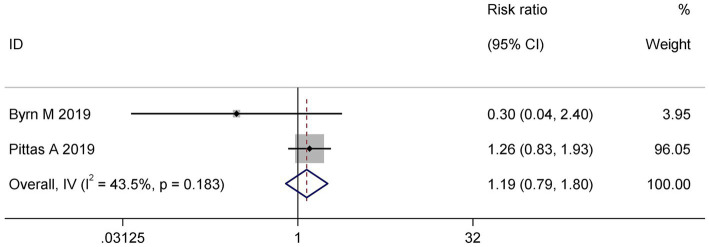
Forest plot of the effect of vitamin D supplementation on HbA1c levels.

Meta-analysis of HbA1c outcomes, derived from four studies (15, 18, 22, 23), demonstrated no statistically significant effect of vitamin D supplementation compared to placebo or control cohorts (SMD: 0.08; 95% CI: −0.25 to 0.37; I^2^ = 86.5%; *p* < 0.001). However, the analysis was characterized by pronounced inter-study heterogeneity (I^2^ = 86.5%, *p* < 0.001), suggesting considerable variability in trial design, population characteristics, or intervention parameters. These findings are depicted in Figure 2.

#### 3.3.2 HOMA-IR

**Figure 3:**
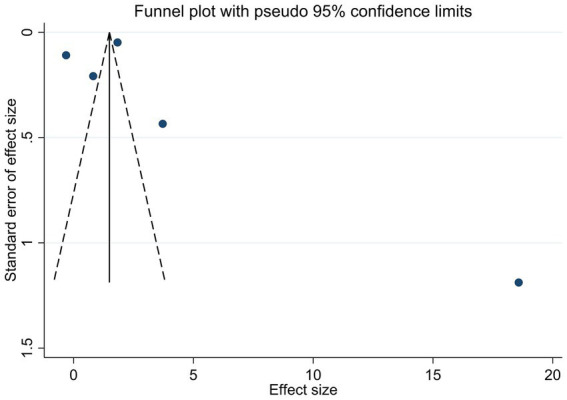
Forest plot of the effect of vitamin D supplementation on HOMA-IR.

Three trials (15, 17, 23) reported HOMA-IR data. Pooled analysis demonstrated no statistically significant effect of vitamin D supplementation on insulin resistance (SMD: 5.95; 95% CI: −2.87 to 14.77; I^2^ = 100.0%; *p* < 0.001) (Figure 3). The extremely high heterogeneity indicates substantial methodological and population-level variability across studies.

#### 3.3.3 Serum 25(OH)D levels

**Figure 4:**
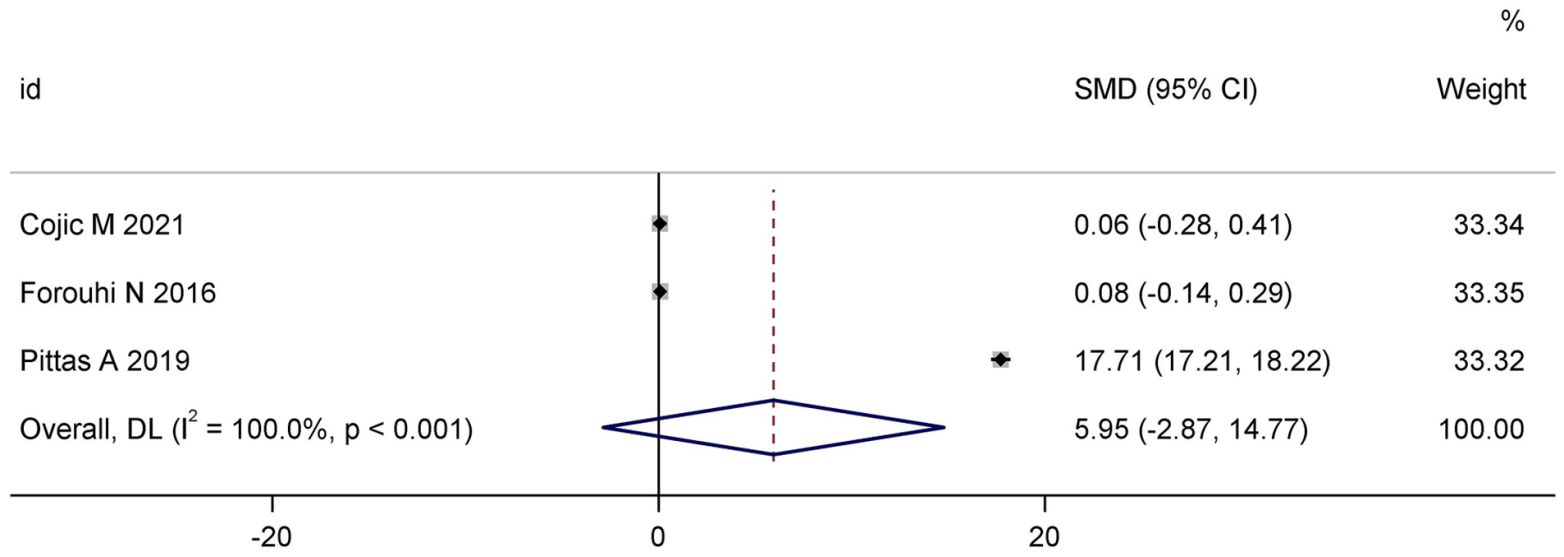
Forest plot of the effect of vitamin D supplementation on serum 25(OH)D levels.

Meta-analysis of five trial (17, 20–23) demonstrated a robust and statistically significant elevation in serum 25(OH)D levels among participants receiving vitamin D supplementation (SMD: 4.01; 95% CI: 2.43 to 5.59; I^2^ = 99.3%; *p* < 0.001) relative to control cohorts. However, heterogeneity was substantial, likely reflecting considerable inter-study variability in supplementation dosage, frequency, formulation, and participants’ baseline vitamin D status (Figure 4).

Despite the robust and statistically significant elevation in serum 25(OH)D levels (SMD: 4.01) following supplementation, this substantial biochemical change did not translate into significant improvements in the co-primary metabolic endpoints of HbA1c or HOMA-IR. The clinical relevance of this isolated 25(OH)D increase is therefore uncertain within the context of glycemic management in T2D, suggesting that achieving serum 25(OH)D sufficiency alone may be insufficient to modify glucose metabolism in this population.

### 3.4 Secondary outcomes

#### 3.4.1 Adverse events

**Figure 5:**
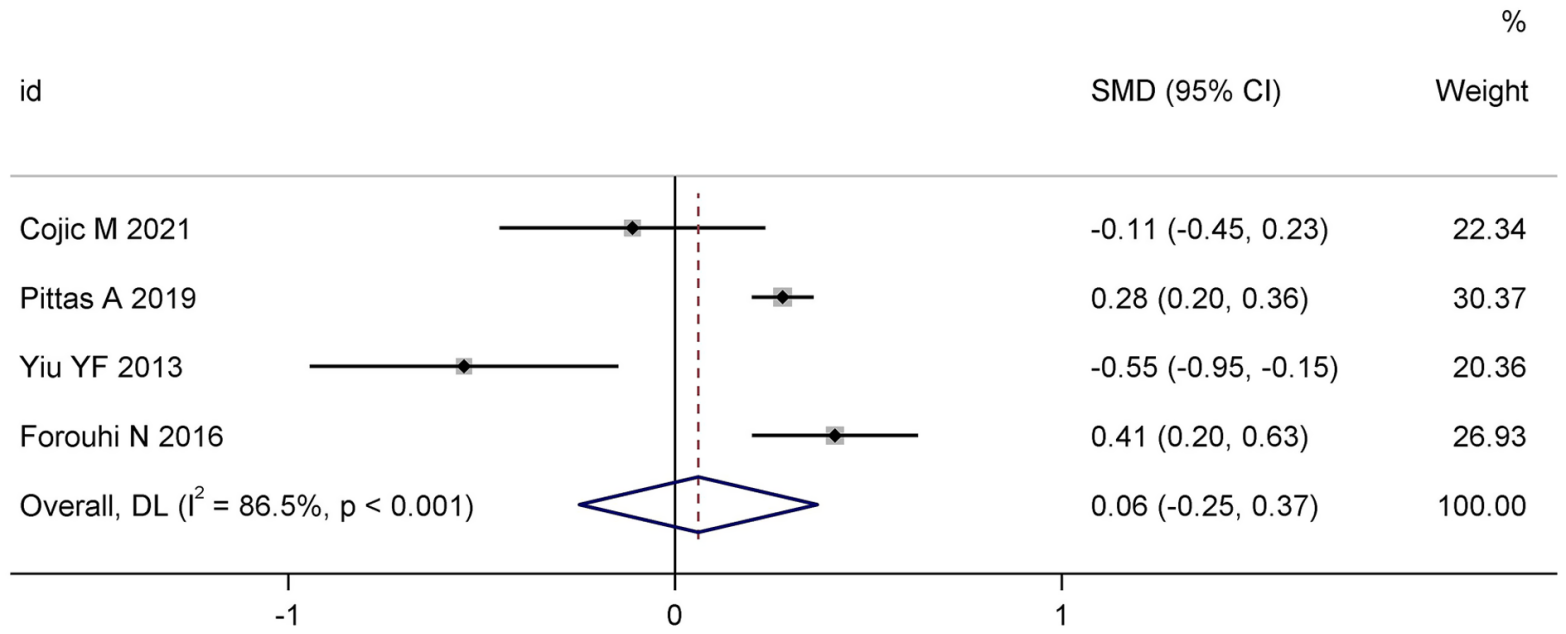
Forest plot of adverse events in vitamin D supplementation versus control groups.

Two trials (16, 17) contributed adverse event data. Meta-analysis revealed no significant difference in adverse event risk between vitamin D and control cohorts (RR: 1.19; 95% CI: 0.79 to 1.80; I^2^ = 43.5%; *p* = 0.183) indicating a comparable safety profile. The moderate heterogeneity observed may reflect differences in event classification or monitoring protocols (Figure 5).

### 3.5 Publication bias

**Figure 6:**
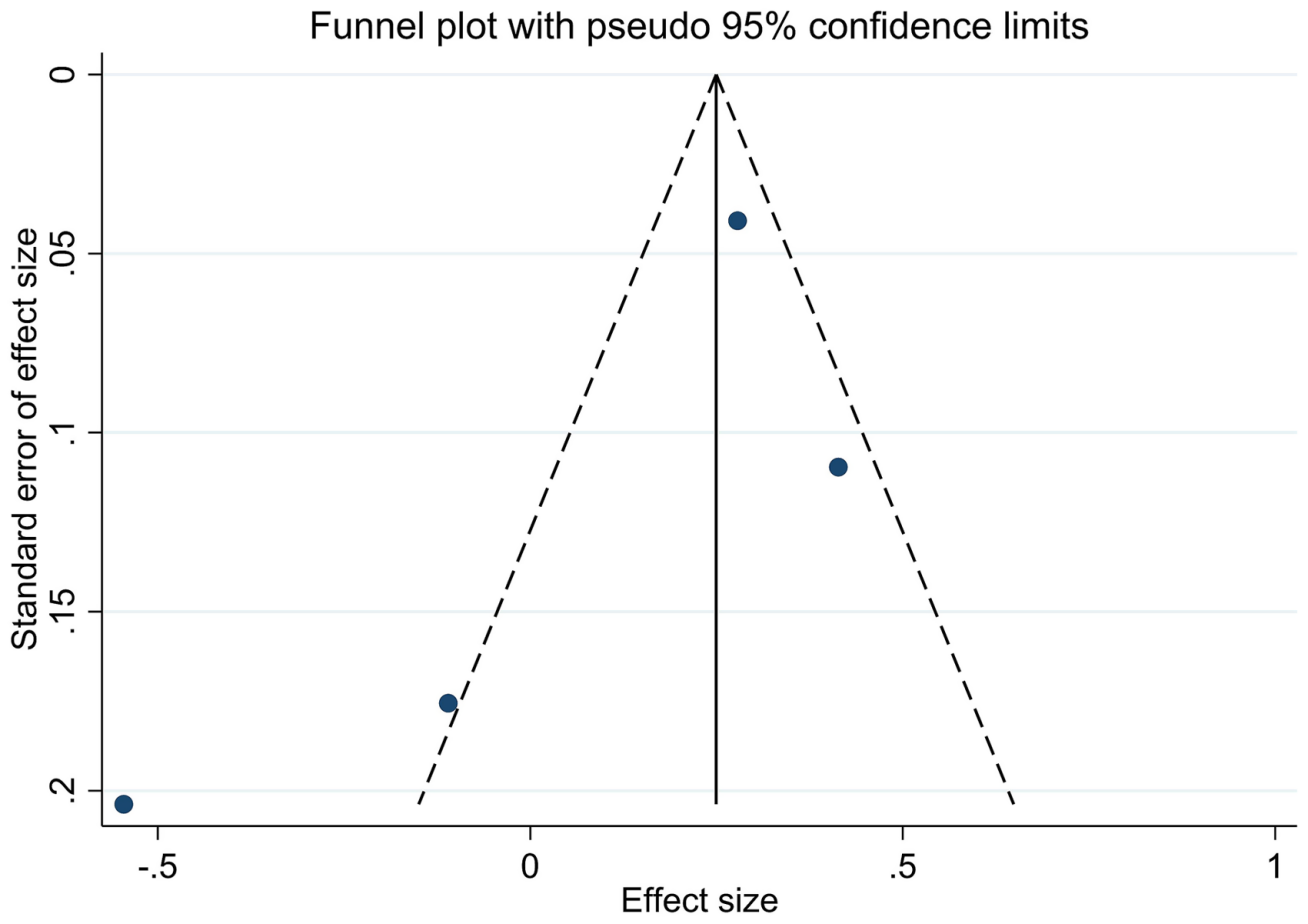
Funnel plot of studies reporting the effect of vitamin D supplementation on HbA1c levels.

**Figure 7:**
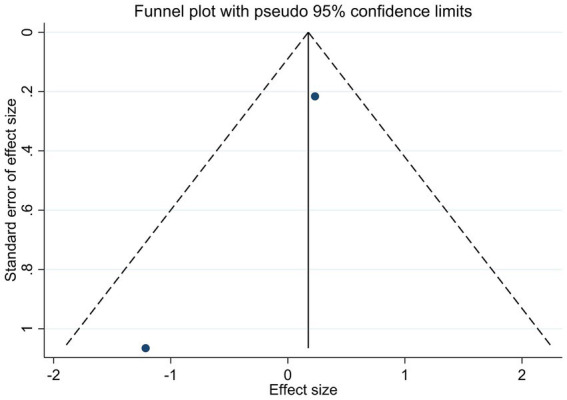
Funnel plot of studies reporting the effect of vitamin D supplementation on serum 25(OH)D levels.

**Figure 8:**
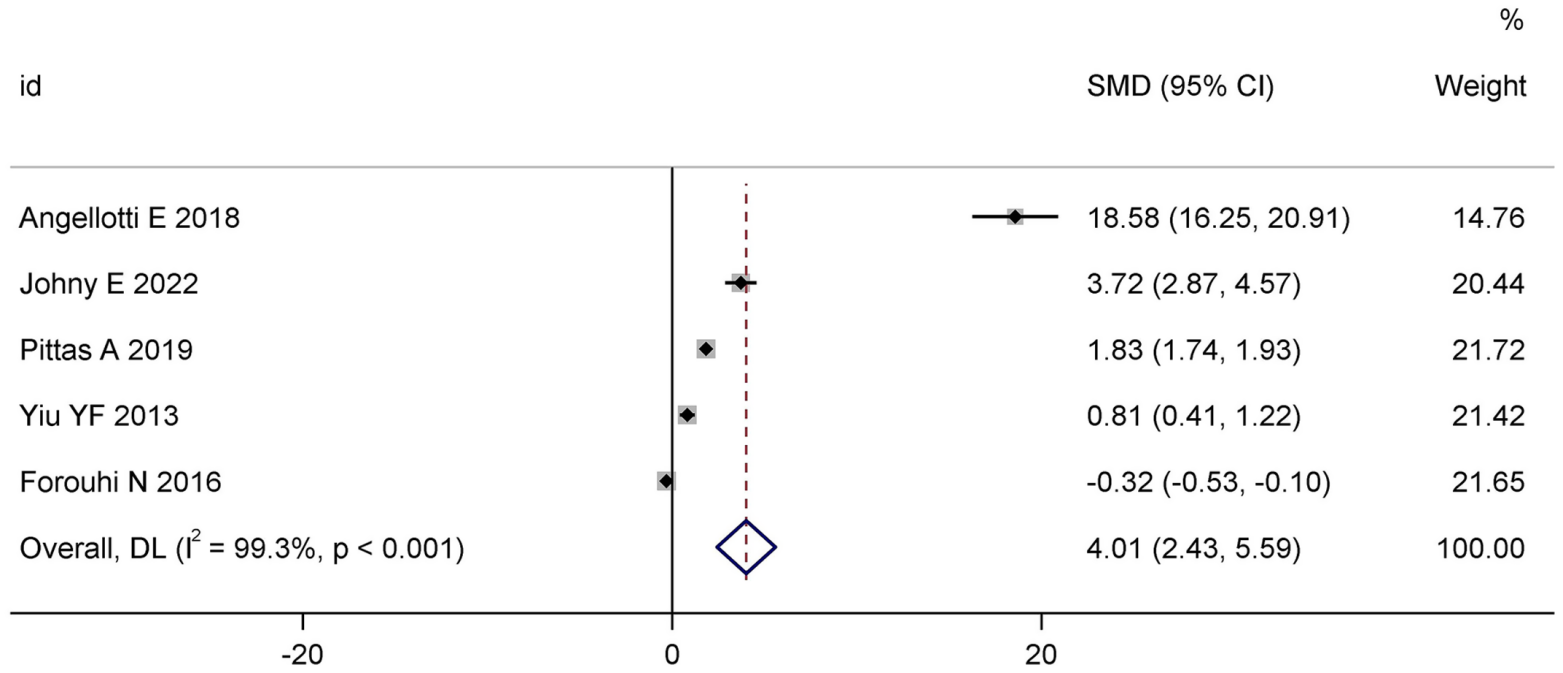
Funnel plot of studies reporting adverse events in vitamin D supplementation versus control groups.

To appraise the potential for publication bias, funnel plots were constructed for key outcomes, HbA1c, serum 25(OH)D levels, and adverse events (Figures 6–8). Qualitative appraisal of plot symmetry, in conjunction with Egger’s regression test, yielded no evidence of significant dissemination bias: HbA1c (*p* = 0.701), serum 25(OH)D (*p* = 0.053), or adverse events (*p* = 0.509).

### 3.6 Exploration of heterogeneity and subgroup analyses

Given the substantial heterogeneity observed in the primary outcomes (HbA1c, HOMA-IR, and 25(OH)D), pre-specified subgroup analyses were conducted where data permitted. For HbA1c, subgrouping by intervention duration (≤6 months vs. >6 months) and vitamin D dosage (<2000 IU/day vs. ≥2000 IU/day) did not reveal significant differences between subgroups, and heterogeneity within subgroups remained high. An analysis stratified by baseline vitamin D status (deficient vs. non-deficient) was planned but deemed unfeasible due to the inconsistent reporting of baseline 25(OH)D levels and the lack of a uniform deficiency threshold across the included trials. The variability in intervention durations (3–12 months) and dosages (500 to 60,000 IU) likely contributed substantially to the observed statistical heterogeneity.

## 4 Discussion

In this comprehensive review of 10 randomized controlled experiments (RCTs) involving a cumulative cohort of 3,460 type 2 diabetes (T2D) individuals diagnosed with type 2 diabetes mellitus (T2DM), we assessed the therapeutic efficacy and safety profile of vitamin D supplementation in modulating glycemic outcomes. While the intervention consistently elevated vitamin D concentrations, it did not confer significant improvements in markers of glucose regulation or insulin sensitivity. Notably, the safety analysis revealed no increase in the incidence of adverse events relative to placebo or standard therapeutic regimens, underscoring the intervention’s favorable tolerability.

Our findings contribute to an evolving body of meta-analytical evidence on this topic. While our conclusion that vitamin D supplementation does not significantly improve HbA1c aligns with several earlier reviews (25, 26), our analysis offers distinct advancements. First, compared to the umbrella of interventional meta-analyses by Musazadeh et al. (27), which summarized the effects across multiple biomarkers, our study provides a more focused and updated pooled quantification specifically for core glycemic indices (HbA1c and HOMA-IR) in T2D. Second, in contrast to the systematic review by Max et al. (28) which focused specifically on high-dose vitamin D, our inclusion of a broader dosing range (500 to 60,000 IU) enhances the generalizability of our null findings across common clinical practices. Finally, and most importantly, our updated search captured the most recent evidence up to mid-2024, including the large-scale trial by Pittas et al. (17), thereby providing a more contemporary and comprehensive synthesis than some prior analyses (26).

The absence of significant glycemic benefit, particularly with respect to HbA1c reduction, mirrors findings from select prior meta-analyses (29, 30), though it diverges from others that have reported marginal yet statistically significant effects (31, 32). These divergent outcomes may be attributable to variations in trial design, population characteristics, intervention duration, and analytic strategies. By restricting inclusion to RCTs with a minimum duration of 3 months and focusing specifically on T2D, the present analysis offers a more refined and robust evaluation of the long-term efficacy of vitamin D augmentation within this specific clinical context.

The observed increase in serum 25(OH)D levels substantiates the efficacy of the supplementation regimens in correcting vitamin D insufficiency. Nonetheless, this biochemical improvement failed to elicit significant changes in glycemic control or insulin resistance. The apparent dissociation between improved 25(OH)D levels and lack of metabolic benefits prompts critical reconsideration of the mechanistic pathways hypothesized to underlie vitamin D’s role in glucose homeostasis among individuals with T2DM.

The absence of significant improvements in glycemic control and insulin resistance may be attributed to multiple interrelated factors. Chief among them is the unresolved question of the optimal serum 25(OH)D threshold necessary to elicit metabolic benefits. Although supplementation protocols reliably increased circulating 25(OH)D levels, the levels achieved may have fallen short of the physiological threshold required to impact glucose homeostasis. Although supplementation protocols reliably increased circulating 25(OH)D concentrations, the levels achieved may have fallen short of the physiological threshold required to impact glucose homeostasis. Notably, some investigators posit that serum 25(OH)D concentrations above 75–80 nmol/L are essential for optimizing glucose metabolism (33), a benchmark not uniformly reached across the included trials.

A potential explanation for the absence of significant metabolic improvements lies in the relatively short duration of intervention across the included studies, which ranged from 3 to 12 months. Such timeframes may be insufficient to capture clinically meaningful changes in glycemic indices especially in HbA1c, a biomarker that reflects mean glycemia over the prior two to 3 months. Longer-duration trials may be warranted to more definitively ascertain the therapeutic potential of vitamin D supplementation in T2D.

Moreover, the heterogeneity in vitamin D supplementation protocols among the included trials may have influenced the observed variability in outcomes. Dosage and administration frequency varied considerably, with regimens ranging from daily to monthly dosing. Such differences in pharmacokinetics and bioavailability may yield distinct effects on glucose metabolism and insulin sensitivity. Accordingly, future investigations should prioritize the delineation of an optimal dosing strategy to maximize potential metabolic benefits in patients with type 2 diabetes.

The baseline vitamin D status of participants may have significantly modulated the physiological response to supplementation. Several studies suggest that the therapeutic benefits of vitamin D are more evident among individuals with baseline insufficiency or deficiency (34). However, the inconsistent documentation of initial 25(OH)D levels and the lack of standardized thresholds for defining deficiency across trials impeded the ability to perform robust subgroup analyses stratified by baseline vitamin D levels.

Genetic variability may further contribute to the heterogeneity in response to vitamin D augmentation. Specifically, polymorphisms in the vitamin D receptor (VDR) gene, as well as in genes involved in the metabolic pathways of vitamin D catabolism, have been associated with differential physiological responses to supplementation and susceptibility to T2DM (35).

The collective findings from this meta-analysis do not support the routine use of vitamin D supplementation for the purpose of improving glycemic control or insulin resistance in the general T2D population. However, given its favorable safety profile and established benefits for bone health, supplementation remains a reasonable intervention for individuals with T2D who have confirmed vitamin D deficiency or insufficiency. For clinicians, the key takeaway is to prioritize the correction of deficiency where it exists, rather than prescribing vitamin D as a primary or adjuvant glucose-lowering therapy for all patients with T2D.

Although no significant improvements were observed in the primary metabolic endpoints, our analysis affirms the overall safety and tolerability of vitamin D supplementation. The frequency of adverse events was similar between intervention and control groups, with reported effects largely characterized as mild and self-limiting. These finding are consistent with prior clinical trials and meta-analyses, reinforcing the safety profile of vitamin D supplementation at the doses administered in the included studies (36).

Notably, while our comprehensive analysis did not reveal statistically significant improvements in the primary endpoints, select individual studies reported beneficial effects on secondary outcome measures. For instance, Cojic et al. (15) documented improvements in oxidative stress markers, and Shab-Bidar et al. (19) identified reductions in inflammatory biomarkers. These findings imply that vitamin D supplementation may elicit nuanced or targeted effects that are not adequately captured by conventional glycemic indices. Future research should aim to elucidate these potential benefits through the use of refined and mechanistically informative biomarkers.

The null metabolic effect of vitamin D supplementation observed in our study underscores the multifactorial nature of T2D, which involves complex pathophysiological mechanisms such as chronic inflammation (37), intestinal microbiota dysbiosis (38), and organ-specific cell death mechanisms like ferroptosis (39). The management of T2D and its complications often requires a multi-targeted approach, as evidenced by research on combination therapies for conditions like diabetic nephropathy (40). In this complex clinical landscape, our findings suggest that vitamin D monotherapy is not a panacea for glycemic control. Future studies should investigate whether vitamin D can act as a beneficial adjunct within these broader, multi-faceted treatment strategies, particularly for patients exhibiting specific inflammatory phenotypes or metabolic disturbances in pathways such as glutamine metabolism (41).

The present meta-analysis is underpinned by its strict inclusion of randomized controlled trials, an exhaustive search of multiple databases, and a robust sample size. We systemically assessed a wide spectrum of glycemic and safety endpoints, enhancing the comprehensiveness of our evaluation.

Notwithstanding the rigorous methodology, our findings must be interpreted in the context of several limitations. First, substantial heterogeneity was observed across the primary outcomes, which, despite the use of a random-effects model and exploratory subgroup analyses, could not be fully resolved. This variability stems from fundamental differences in trial design, participant characteristics, and intervention protocols. Second, the relatively short duration (≤12 months) of most included trials may be insufficient to capture long-term metabolic effects, particularly on a chronic condition like T2D. Third, the influence of key confounding factors, such as body mass index (obesity), sunlight exposure, physical activity, and the use of concomitant glucose-lowering or lipid-lowering medications, could not be accounted for in our pooled analysis and may have modulated the observed outcomes. Fourth, while we attempted to analyze the impact of baseline vitamin D status, this was hampered by inconsistent reporting and the lack of standardized deficiency thresholds, rendering our conclusions on this moderator inherently observational and hypothesis-generating. Finally, although our search was inclusive of Chinese databases, the restriction to English and Chinese publications may have introduced a language bias, and the small sample sizes of some trials may limit the generalizability of their individual findings.

Future research should move beyond unselected T2D populations and focus on targeted approaches. Priority should be given to large-scale, long-term RCTs specifically designed for T2D patients with baseline vitamin D deficiency, employing standardized, high-dose supplementation regimens to achieve and maintain sufficiency. Furthermore, studies should incorporate comprehensive biomarker panels to explore effects on inflammation, oxidative stress, and endothelial function, which may be more sensitive to vitamin D intervention than conventional glycemic indices. Investigating the interaction between vitamin D supplementation and genetic polymorphisms, as well as its effects in specific patient phenotypes (e.g., those with high insulin resistance or significant obesity), could also help identify potential responders. Additionally, exploring the potential of combination therapies—such as integrating vitamin D with other nutritional agents or treatment strategies that target related metabolic pathways, an area informed by research into traditional medicine and novel drug delivery systems (42–45)—may unlock synergistic effects and provide a more effective approach to managing the complex pathophysiology of T2D.

## 5 Conclusion

In conclusion, this meta-analysis demonstrates that vitamin D supplementation consistently elevates serum 25(OH)D concentrations in individuals with type 2 diabetes but does not confer significant benefits for glycemic control or insulin resistance. The intervention was safe and well-tolerated at the studied doses. Therefore, routine vitamin D supplementation cannot be recommended solely for the purpose of improving metabolic outcomes in unselected T2D populations.

Future research should prioritize identifying specific subpopulations most likely to benefit, particularly those with baseline vitamin D deficiency, and should employ standardized, long-term dosing protocols to definitively assess its therapeutic potential. These targeted investigations are crucial to translate the observed biochemical efficacy of vitamin D into meaningful clinical benefits.

## Data Availability

The original contributions presented in the study are included in the article/supplementary material, further inquiries can be directed to the corresponding author.
